# EsrE-A *yigP* Locus-Encoded Transcript-Is a 3′ UTR sRNA Involved in the Respiratory Chain of *E. coli*

**DOI:** 10.3389/fmicb.2017.01658

**Published:** 2017-08-29

**Authors:** Hui Xia, Xichen Yang, Qiongwei Tang, Jiang Ye, Haizhen Wu, Huizhan Zhang

**Affiliations:** ^1^State Key Laboratory of Bioreactor Engineering, East China University of Science and Technology Shanghai, China; ^2^Department of Applied Biology, East China University of Science and Technology Shanghai, China

**Keywords:** *yigP* locus, aerobic growth, Q_8_, EsrE sRNA, succinate dehydrogenase, *E. coli*

## Abstract

The *yigP* locus is widely conserved among γ-proteobacteria. Mutation of the *yigP* locus impacts aerobic growth of Gram-negative bacteria. However, the underlying mechanism of how the *yigP* locus influences aerobic growth remains largely unknown. Here, we demonstrated that the *yigP* locus in *Escherichia coli* encodes two transcripts; the mRNA of ubiquinone biosynthesis protein, UbiJ, and the 3′ untranslated region small regulatory RNA (sRNA), EsrE. EsrE is an independent transcript that is transcribed using an internal promoter of the *yigP* locus. Surprisingly, we found that both the EsrE sRNA and UbiJ protein were required for Q_8_ biosynthesis, and were sufficient to rescue the growth defect ascribed to deletion of the *yigP* locus. Moreover, our data showed that EsrE targeted multiple mRNAs involved in several cellular processes including murein biosynthesis and the tricarboxylic acid cycle. Among these targets, *sdhD* mRNA that encodes one subunit of succinate dehydrogenase (SDH), was significantly activated. Our findings provided an insight into the important function of EsrE in bacterial adaptation to various environments, as well as coordinating different aspects of bacterial physiology.

## Introduction

Gene regulation, which occurs at multiple levels including transcriptional and post-translational control, is a vital mechanism across all domains of life (Bobrovskyy and Vanderpool, 2013). One emerging class of post-transcriptional regulators in bacteria is small regulatory RNAs (sRNAs; Bobrovskyy and Vanderpool, 2013). Previous studies suggest that sRNAs are typically short transcripts that vary from 50 to 500 nucleotides and rarely contain an open reading frame (ORF; Bobrovskyy and Vanderpool, 2013). sRNAs act by perfect or limited base-pairing to target mRNAs to influence their stability or/and translation (Updegrove et al., 2015). In γ-proteobacteria, most sRNAs require the RNA chaperone, Hfq, to stabilize themselves and promote sRNA–mRNA intermolecular interaction (Chao and Vogel, 2010; Vogel and Luisi, 2011). Such interaction principally inhibits the translation and/or induces the degradation of their target mRNAs in a ribonuclease E (RNase E)-dependent manner (Masse and Gottesman, 2002; Masse et al., 2003; Morita et al., 2005; Frohlich and Vogel, 2009). However, compared to their negative effect on gene expression, the positive regulation by sRNAs remains largely unknown (Papenfort and Vanderpool, 2015). Besides the most characterized intergenic sRNAs, recent RNA-seq studies suggest bacterial sRNA-encoding genes are also located in coding regions (Lalaouna et al., 2015; Miyakoshi et al., 2015a). The 3′ untranslated region (UTR) sRNAs belong to a newly characterized non-intergenic class. They are produced by either mRNA processing or transcription from independent promoters within genes. In both cases, the 3′ UTR sRNAs share the same sequences with the 3′ regions of the coding genes (Miyakoshi et al., 2015b). Therefore, these 3′ UTR sRNAs pose a challenge for ongoing gene or locus function characterization.

All 11 *ubi* genes (*ubiA–J* and *ubiX*) are required for the biosynthesis of Q_8_ in *Escherichia coli*. Most of their products catalyze specific reactions in the biosynthetic process, starting from chorismate (Van Beilen and Hellingwerf, 2016). The *yigP* (renamed *ubiJ*) locus is highly conserved among almost 80 organisms, many of which are γ-proteobacteria. In *E. coli*, *yigP* is located between *ubiE* and *ubiB* loci, and may be synchronously transcribed with them under an upstream promoter of *ubiE* (Poon et al., 2000; Aussel et al., 2014a). UbiE is well-known for the C methylation reactions in both Q_8_ and menaquinone (MK8) biosynthesis (Aussel et al., 2014a). A mammalian homolog of UbiB, COQ8A (ADCK3), was recently found to display ATPase activity and interacted with lipid CoQ intermediates (Stefely et al., 2016). However, though *yigP* mutant strains of *Salmonella* and *E. coli* showed significant defects in Q_8_ biosynthesis (Aussel et al., 2014a), the underlying mechanism remains unrevealed.

Our previous studies found that the 3′ region of *yigP* transcribed an sRNA named EsrE with indispensable function in *E. coli* (Chen et al., 2012). A recent study in *Salmonella* found that the *yigP* locus encoded a protein named UbiJ required for Q_8_ biosynthesis. Based on evidence that a “scrambled mutation” of the 3′ region of *Salmonella ubiJ* had no phenotype, it was concluded that the aerobic growth-promoting function of the *yigP* locus was mediated by UbiJ but not the sRNA, EsrE (Aussel et al., 2014a). In this work, we provide new evidence that the *yigP* locus contains two genes, *ubiJ* and *esrE. UbiJ* encoded a protein, UbiJ, while *esrE* transcribed an sRNA, EsrE. Furthermore, our results showed that besides UbiJ, the sRNA EsrE was also critical for the aerobic growth of *E. coli* and required for Q_8_ biosynthesis. In addition, we found that EsrE might maintain cellular processes of *E. coli* by activating multiple mRNAs.

## Materials and Methods

### Bacterial Strains and Growth Conditions

The wild-type is *E. coli* K-12 strain JM83. Unless otherwise stated, the cells were grown at 37°C in liquid or on solid Luria-Bertani (LB) media supplemented with chloramphenicol (30 μg/mL) or ampicillin (100 μg/mL) as indicated. All strains and plasmids used here are listed in Supplementary Table S1.

### Inactivation of the *yigP* Locus

The λ Red-mediated recombination method was used to delete different regions of the *yigP* locus with an insert of apramycin resistance cassette. The mutant fragments were transferred to *E. coli* JM83 by electroporation.

### Construction of a 3 × Flag-*yigP*-Fused *E. coli* Strain

To carry out an *E. coli* strain that expresses a fused YigP protein with a C-terminal [Gly_4_Ser]_3_ linker plus a triple-Flag tag (DYKDHDGDYKDHDIDYKDDDDK), a pMAK705-derived temperature-sensitive plasmid, pMAK-yigP-flag, was created using an approach described previously (Bush et al., 2013). Using the pMAK-yigP-flag according to previous methods (Chen et al., 2012), the chromosomal *yigP* insertion strain, WT::*flag*, was constructed.

### Quinone Extraction and Analysis

The extraction and analysis of quinones was performed as described by Ozeir et al. (2015) and Van Beilen and Hellingwerf (2016). The cells were cultured for 10 h before collection by centrifugation, washed twice with distilled water, and their wet weights determined. A total of 150 μL of 0.15 M KCl (a Q_10_ solution used as an internal standard, 100 μg/mL in ethyl alcohol, 0.863 μL/mg of wet weight) and 0.6 mL of methanol were added to cell pellets, and the tubes vortexed for 15 min. Then 0.4 mL of petroleum ether (boiling range, 40–60°C) was added and the tubes vortexed for 5 min. The phases were separated by centrifugation at 5000 rpm at room temperature for 5 min. The upper petroleum ether layer was transferred to a fresh tube. Petroleum ether (0.4 mL) was added to the methanol-containing tube, and the extraction was repeated once more. The petroleum ether layers were combined and dried. The lipids were resuspended in 100 μL of ethyl alcohol.

The samples were fractionated with high-performance liquid chromatography (HPLC) using a reversed-phase C18 column (Betabasic-18, 5 μm, 4.6 mm × 250 mm, Agilent Technologies). The column was equilibrated with pure ethyl alcohol as the mobile phase at a flow rate of 1 mL/min. Detection of quinones was performed using an UV/Vis absorption detector at 275 nm for ubiquinone (UQ).

### Real-time qRT-PCR

Isolation of bacterial total RNA was performed with High Pure RNA Isolation Kit (Roche Diagnostics, Germany) according to the manufacturer’s instructions. Total RNA was the template for qRT-PCR reaction using Reverse Transcription M-MLV (RNase H-) kit (TaKaRa, Japan) and SYBR Green PCR Master Mix (TOYOBO, Japan). The primers for qRT-PCR are listed in Supplementary Table S2. The levels of gene expressions were normalized using the *rpoD* transcript data.

### SDS-PAGE and Western Blot Analysis

To investigate whether the *yigP* locus encodes a protein, SDS-PAGE and western blotting were performed. All the strains were grown to an indicated time in LB media, and the cells were washed with phosphate-buffered saline. The samples were sonicated and total protein quantified following standard protocols. The protein was denatured at 100°C for 5 min. Total protein from each sample was loaded for SDS-PAGE, and western blotting was performed with Flag-tag primary and horseradish-peroxidase-conjugated goat anti-mouse secondary (AOGMA, United States) antibodies.

### β-Galactosidase Assays

Cells were cultured overnight and then subcultured at 1:100 in fresh LB. After growing for 5 h to an optical density (OD)_600_ of 2, equivalent cell densities were collected, pelleted, and permeabilized by ultrasonication. Ortho-nitrophenyl-β-D-galactopyranoside hydrolysis was determined in triplicate to measure β-galactosidase activity as described previously (Wang et al., 2012).

## Results

### The *yigP* Locus Encodes Two Products: EsrE and UbiJ

**FIGURE 1:**
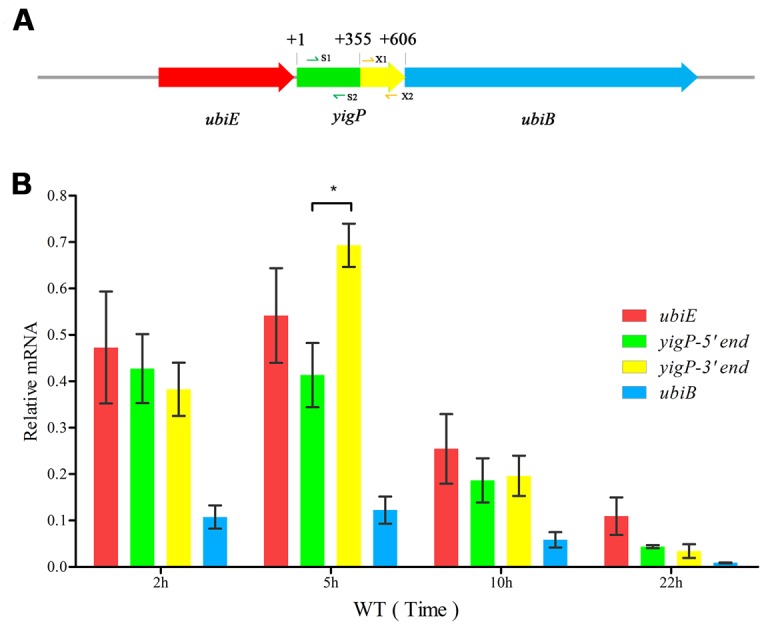
The *yigP* locus transcribes two products. **(A)** Schematic representationof the *ubiE*, *yigP*, and *ubiB* loci in *Escherichia coli*. S1/2 and X1/2 represent primer pairs used to analyze *yigP* expression (yigP-S1/2 and yigP-X1/2). **(B)** qRT-PCR analysis of the *yigP* locus transcripts in wild type strain at different time points. Bacteria were grown in triplicate in Luria-Bertani (LB) liquid culture, and equivalent cell densities were collected. The reactions were performed in duplicate, and the relative expression changes were calculated using the 2^ΔΔCT^ method with the constitutively expressed *rpoD* gene serving as the endogenous control. ^∗^*P* < 0.05.

The *ubiE*, *yigP*, and *ubiB* loci in *E. coli* are considered an operon (Poon et al., 2000) essential for Q_8_ biosynthesis (Aussel et al., 2014b). However, how *yigP* contributes to this process is still unclear. In our previous study, we found that the 3′ region of *yigP* encoded a small RNA called EsrE that might have essential functions in *E. coli* (Chen et al., 2012). Recently, the *yigP* locus was reported to encode a protein called UbiJ that is required for Q_8_ biosynthesis and aerobic growth in *Salmonella* and *E. coli* (Aussel et al., 2014a). To further determine whether *yigP* locus has two transcripts, we designed two pairs of primers, yigP-S1/2 and yigP-X1/2 targeting its 5′ and 3′ regions, respectively, for transcript analysis (**Figure 1A**). Because *ubiE*, *yigP*, and *ubiB* loci are considered to form an operon (Poon et al., 2000), *ubiE* and *ubiB* mRNAs were also examined (**Figure 1B**). As shown by qRT-PCR, *ubiJ* mRNA was expressed throughout the lifetime of the bacteria (**Figure 1B**). In contrast, *esrE* expression was only detected during the late exponential growth phase (approximately 5 h) as indicated by the finding that the transcript from the 3′ region was more abundant than the transcript from the 5′ region at 5 h (**Figure 1B**). These observations suggested that UbiJ was the constitutively expressed product of the *yigP* locus, while EsrE was transiently generated during the transition to the stationary phase.

**FIGURE 2:**
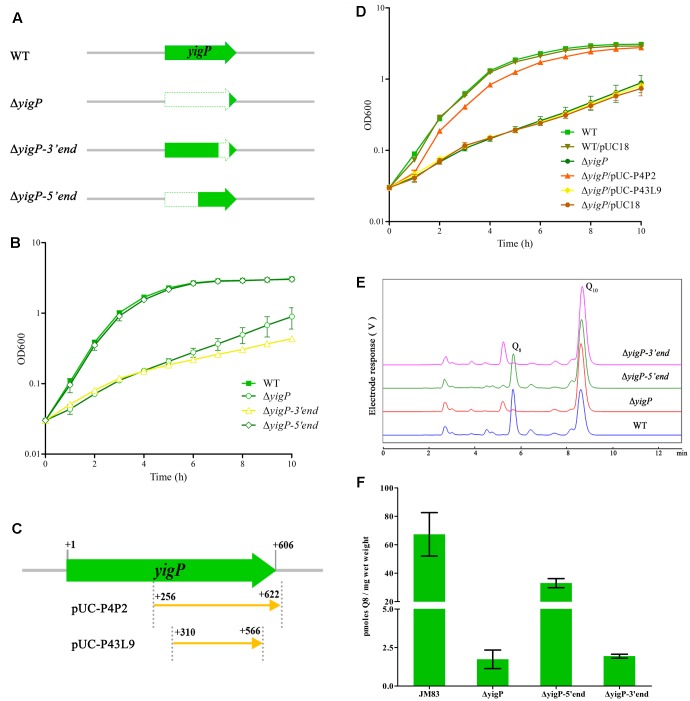
Functional regions of the *yigP* locus. **(A)** Schematic representation of the different *yigP* locus mutants used in this study. Δ*yigP* represents the 572 bp *yigP* deletion mutant, Δ*yigP-3′end* represents the mutant in which 153 bp of the 3′ end of the *yigP* locus was deleted, and Δ*yigP-5′end* represents the mutant in which 283 bp of the 5′ end of the *yigP* locus was deleted. **(B)** The results of a representative experiment are shown. Wild type (filled squares), Δ*yigP* (open circles), Δ*yigP-3′end* (open triangles), and Δ*yigP-5′end* (open rhombuses) strains were grown overnight and then diluted to an OD_600_ of 0.03 in LB medium at 37°C. Growth was monitored at 600 nm. **(C)** Schematic representation of the *yigP* plasmids used in this work. pUC-P4P2 and P43L9 represent the plasmids carrying the 367 and 267 nucleotides, respectively, located at the 3′ end of *yigP*. **(D)** Wild type (filled squares), wild type transformed with pUC18 (filled inverted triangles), Δ*yigP* (filled circles) and Δ*yigP* strains transformed with pUC-P4P2 (filled triangles), P43L9 (filled rhombuses), and pUC18 (filled hexagons) were grown overnight and then diluted to an OD_600_ of 0.03 in LB. Growth was monitored at 600 nm. The experiment was performed at least three times, and identical patterns were obtained. **(E)** High-performance liquid chromatography (HPLC) separation of the strains described for panel **(A)** with the eluate analyzed with A_275_. The identified quinones, Q_8_ and Q_10_, are indicated. **(F)** Quantification of cellular Q_8_ content (*n* = 3) of the same cells as in panel **(A)** in picomoles per milligram of wet weight. Error bars represent standard deviation.

In order to determine the contribution of the two transcripts to aerobic growth, we constructed three deletion mutants containing different regions of the *E. coli yigP* locus (**Figure 2A**). All mutants were successfully obtained. As shown in Supplementary Figure S1, the colonies of the full region (Δ*yigP*) and 3′ region (Δ*yigP-3′end*) mutant strains that grew on LB plates under aerobic conditions were much smaller than the colonies of the 5′ region mutant strain (Δ*yigP-5′end*) (Supplementary Figure S1). These mutant strains and the wild type strain were then cultured in LB under aerobic conditions. Consistent with our previous results, Δ*yigP* and Δ*yigP-3′end* strains exhibited significant growth defects compared to the wild type strain. In contrast, Δ*yigP-5′end* strain grew just as well as the wild type strain (**Figure 2B**). Because the *yigP* locus was truncated in Δ*yigP-5′end* strain to prevent UbiJ translation *in vivo*, our results suggest EsrE was the product of the *yigP* locus that maintained the aerobic growth of *E. coli.*

To further determine the functional region of the *yigP* locus, we transformed two plasmids containing different truncated fragments (Chen et al., 2012) in proximity to the *esrE*-coding region into the Δ*yigP* strain (**Figure 2C**). The plasmid carrying the 367 bp whole *esrE*-coding sequence fragment partially (P4P2) restored the growth defect of Δ*yigP* strain, whereas the 267 bp partial *esrE*-coding sequence fragment (P43L9) failed to do so (**Figure 2D**). Because the *P_lacZ_* promoter located in pUC18 had been removed, the experiment also revealed that the 3′ region fragment (P4P2) had its own promoter. Hence, the EsrE transcript from an independent promoter located within the *yigP* locus contributed to the growth of *E. coli*.

In addition, the *yigP* locus was previously shown to be essential for Q_8_ biosynthesis, and the amount of Q_8_ was tightly related to the aerobic growth of *E. coli* (Aussel et al., 2014a). We therefore measured the cellular Q_8_ content of the three mutant (**Figure 2A**) and the wild type strains by HPLC analysis (**Figure 2E**) (Ozeir et al., 2015; Van Beilen and Hellingwerf, 2016). Unlike the growth phenotype (**Figure 2B**), the Q_8_ content in Δ*yigP-5′end* strain was 50% of the wild type strain (33 and 67 pmol, respectively) (**Figure 2F**). Reasoning that Δ*yigP-5′end* strain had no UbiJ protein expression *in vivo*, EsrE was responsible for the half amount of Q_8_, which might be enough to maintain normal aerobic growth of *E. coli* (**Figure 2B**; Aussel et al., 2014a,b). Meanwhile, the Δ*yigP* and Δ*yigP-3′end* strains had tiny amounts of Q_8_ (**Figure 2F**), which was in agreement with previous evidence that the *yigP* locus was required for the synthesis of Q_8_. Taken together, these results indicated that both EsrE and UbiJ from the *yigP* locus were required for Q_8_ production in *E. coli.*

### EsrE Is Required for Aerobic Growth of *E. coli* as an sRNA

**FIGURE 3:**
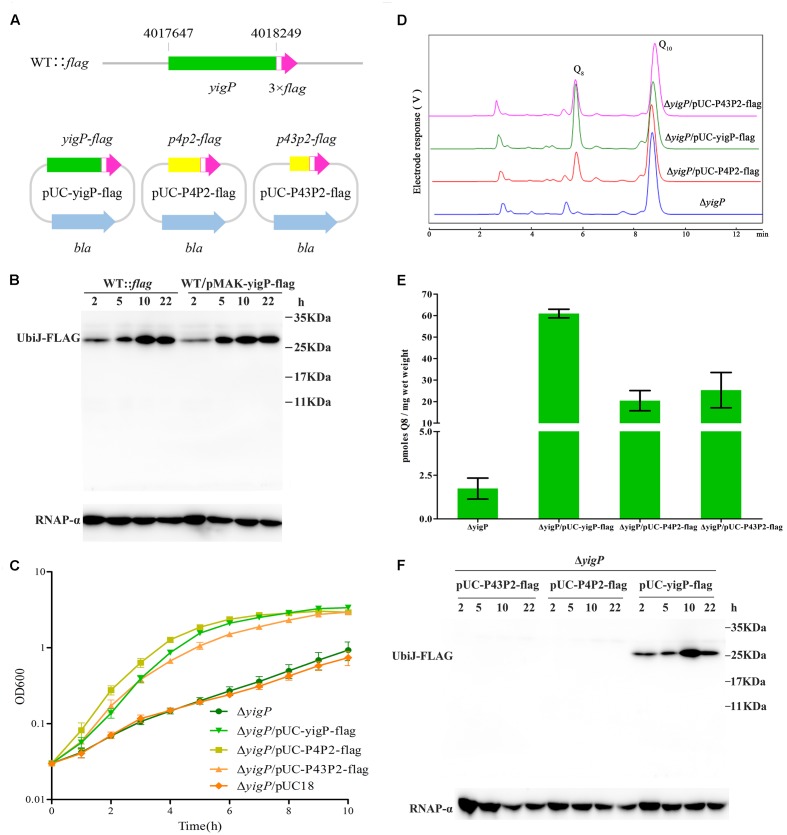
The *yigP* locus encodes EsrE and a 23-kDa protein, UbiJ, both of which have important effects on the growth of *E. coli*. **(A)** Schematic representation of mutant strain, which Linker plus 3 × Flag tag sequences were introduced before the stop codon of the *yigP* locus in wild type bacteria, named WT::*flag*. In addition, Linker plus 3 × Flag tag sequences were introduced before the stop codon of different *yigP* fragments in plasmid pUC18 to yield pUC-yigP-flag, pUC-P4P2-flag, and pUC-P43P2-flag. YigP means the full-length fragment (including 241 bp sequences upstream *yigP* locus, considered as the promoter region of *ubiJ*), P4P2 (367 bp) indicates larger region of *esrE*-coding sequence than P43P2, while P43P2 (313 bp) contains the minimal region of *esrE*-coding sequence. **(B)** Western blot analysis of WT::*flag* and wild type bacteria with pMAK-yigP-flag. The *yigP* variants were separated by 15% SDS-PAGE, blotted onto PVDF membranes and hybridized with a Flag tag antibody or RNAP-α antibody. RNAP-α means RNA polymerase α subunit protein used as a loading control. **(C)** Δ*yigP* (filled circles), Δ*yigP* strains transformed with pUC-yigP-flag (filled inverted triangles), pUC-P4P2-flag (filled squares), pUC-P43P2-flag (filled triangles), and pUC18 (filled rhombuses) were grown overnight and then diluted to an OD_600_ of 0.03 in LB under aerobic conditions. Growth was monitored at 600 nm. The experiment was performed at least three times, and identical patterns were obtained. **(D)** HPLC separation of the strains described for panel **(C)**, except Δ*yigP*/pUC18 with the eluate analyzed with A_275_. The identified quinones, Q_8_ and Q_10_, are indicated. **(E)** Quantification of cellular Q_8_ content (*n* = 3) of the same cells as in panel **(C)**, except Δ*yigP*/pUC18 in picomoles per milligram of wet weight. Error bars represent standard deviation. **(F)** Western blot analysis of pUC-yigP-flag, pUC-P4P2-flag, and pUC-P43P2-flag in Δ*yigP* strain. The *yigP* variants were separated by 15% SDS-PAGE, blotted onto PVDF membranes and hybridized with a Flag tag antibody or RNAP-α antibody. RNAP-α means RNA polymerase α subunit protein used as a loading control.

We investigated whether the 3′ region of the *yigP* locus encodes a polypeptide or only EsrE as sRNA. A 3 × Flag plus Linker coding sequence (111 bp) was chromosomally fused via homologous recombination to the 3′ end of the *yigP* locus in wild type strain using the temperature-sensitive plasmid pMAK-yigP-flag, yielding the WT::*flag* strain (**Figure 3A**). The bacterial cells were harvested from four growth periods, lysed, and the expression levels of the Flag-fused proteins examined by immunoblotting. As expected, a 26 kDa band of UbiJ::Linker-3 × Flag was detected (**Figure 3B**), which coincided with the expression pattern of its mRNA (**Figure 1B**). This finding verified that the *yigP* locus translated the 23 kDa full-length product UbiJ.

Meanwhile, the full-length fragment (including 241 bp sequences upstream of the *yigP* locus, considered as the promoter region of *ubiJ*) and 3′ region P4P2 (367 bp) or P43P2 (313 bp) fragment-fused 3 × Flag plus Linker coding sequence were inserted into pUC18 plasmid without foreign promoter to yield pUC-yigP-flag and pUC-P4P2-flag or pUC-P43P2-flag. These plasmids were then transformed into Δ*yigP* strain. All plasmids rescued the impaired aerobic growth of Δ*yigP* strain (**Figure 3C**). Moreover, by measuring Q_8_ production of the three strains (**Figure 3D**), we found that the Δ*yigP* strain with pUC-yigP-flag could recover Q_8_ amounts to the level of the wild type strain (**Figure 3E**). However, the Q_8_ content in the other two strains only accounted for half of that in the wild type strain (**Figure 3E**), and pUC18 did not affect Q_8_ production in Δ*yigP* strain (Supplementary Figure S2A). This result indicated that both EsrE and UbiJ were important for aerobic growth and Q_8_ biosynthesis. Similarly, only pUC-yigP-flag encoded a 26 kDa protein, while both pUC-P4P2-flag and pUC-P43P2-flag did not present any band by immunoblotting (**Figure 3F**). Meanwhile, all three plasmids transcribed *esrE* as determined by qRT-PCR (Supplementary Figure S2B). These results suggested that the function of the 3′ region fragment relied on an sRNA instead of a polypeptide. Collectively, our results showed that there were two products encoded by different regions of the *yigP* locus: one was the RNA, EsrE, and the other was the 23 kDa protein, UbiJ.

**FIGURE 4:**
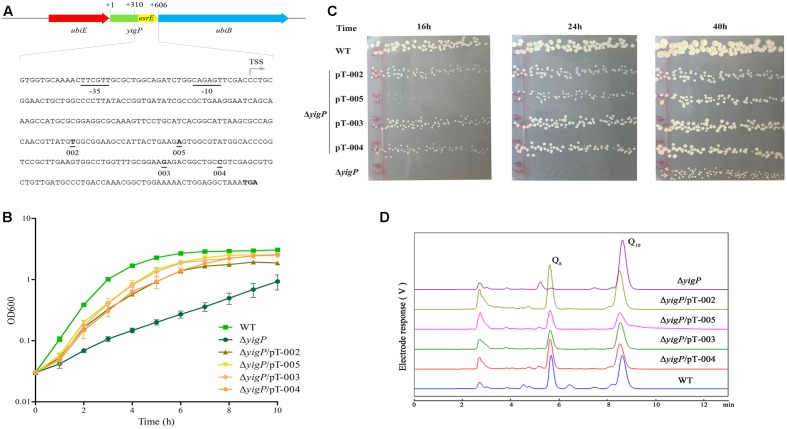
EsrE is a 3′ UTR sRNA. **(A)** Schematic representation and sequence analysis of EsrE. The bent arrow marks the transcription start site (TSS), and the four frame-shift mutation sites in this study are indicated. **(B)** Wild type (filled squares), Δ*yigP* (filled circles), and Δ*yigP* strains transformed with pT-002 (filled triangles), pT-003 (filled rhombuses), pT-004 (filled hexagons), and pT-005 (filled inverted triangles) were grown overnight and then diluted to an OD_600_ of 0.03 in LB. Growth was monitored at 600 nm. The experiment was performed at least three times, and identical patterns were obtained. **(C)** Wild type, Δ*yigP*, and Δ*yigP* strains transformed with pT-002, pT-003, pT-004, and pT-005 were grown on LB plates for 16, 24, and 40 h at 37°C. **(D)** HPLC separation of the strains described for panel **(B)** with the eluate analyzed with A_275_. The identified quinones, Q_8_ and Q_10_, are indicated.

To further examine the role of EsrE, we employed four different P43P2 frameshift mutant fragments (**Figure 4A**). The Δ*yigP* strain was transformed with four plasmids, named pT-002, pT-003, pT-004, and pT-005 (Chen et al., 2012) that contained these mutant fragments. All plasmids significantly complemented the impaired aerobic growth of Δ*yigP* strain when cultured in LB (**Figure 4B**). It is worth mentioning that the Δ*yigP* strain with pT-005 was much smaller than the others when grown on LB plates for up to 24 h, but then reached similar size after 40 h of culture (**Figure 4C**). This indicated that the deletion of site 005 (adenine) might have partly affected the rescue capability of the P43P2 fragment. Meanwhile, we also checked the cellular Q_8_ content of the four strains. All four strains harbored enough amounts of Q_8_ (**Figure 4D**) to recover the growth defect. Overall, because the four mutation sites could affect all potential ORFs, their rescue capabilities provided strong evidence that all the potential ORFs were immune to frameshift mutations, even though these mutant fragments could not recover the growth defect to the same level as the P43P2 fragment (Supplementary Figure S3). Taken together, these data showed that the *esrE* gene located in the 3′ region of the *yigP* locus encoded a 3′ UTR sRNA required for *E. coli* aerobic growth and biosynthesis of Q_8_.

### EsrE Targets Multiple mRNAs *In Vivo*

To uncover the physiological role of EsrE, we tried to search its regulons. Because most sRNAs target mRNAs to regulate their expression levels via perfect or limited base-pairing, the potential regulons of EsrE were screened with two comparative prediction algorithms for sRNA targets, CopraRNA (Wright et al., 2014) and TargetRNA (Kery et al., 2014). Twenty-four candidates were chosen for validation *in vivo* (Supplementary Table S3).

**FIGURE 5:**
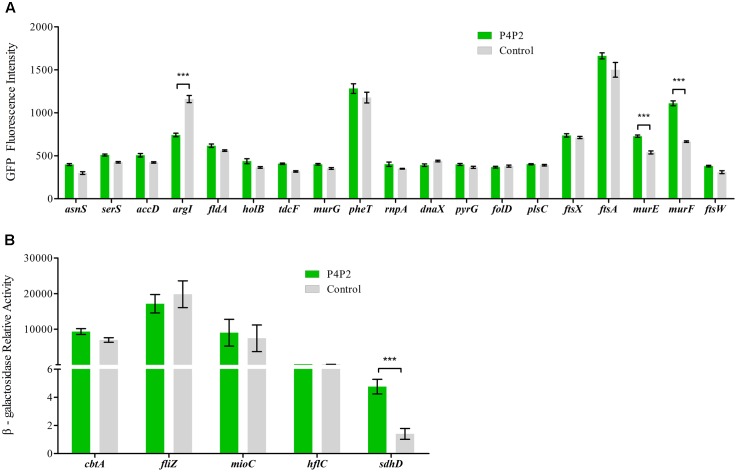
Experimental validation of EsrE target predictions. **(A)** Fluorescence of wild type bacteria carrying the indicated *gfp* fusions and EsrE plasmid (pT-P4P2) or control plasmid (pT-NS). The bacteria were grown in triplicate in LB liquid culture and measured at the indicated growth phase (at 5 h, about OD_600_ of 2). The fluorescence values were given in arbitrary units and corrected for basal fluorescence of a wild type strain harboring plasmid pXG-0. **(B)** Relative activity of β-galactosidase in wild type bacteria carrying the indicated *lacZ* fusions and EsrE (pT-P4P2) or control (pT-NS) plasmid. The bacteria were grown in triplicate in LB liquid culture and were collected at the indicated growth phase (at 5 h, about OD_600_ of 2). β-Galactosidase assays were then performed according to a protocol described previously (Wang et al., 2012). ^∗∗∗^*P* < 0.001.

To further verify these candidates, translational fusions were generated by integrating the 5′ UTRs in-frame with the first codon of *gfp* or *lacZ* (the fluorescence of some GFP fusions are not high enough for detection) and downstream of the heterologous *P_LtetO_* promoter (Supplementary Table S3) (Urban and Vogel, 2007). Wild type cells were then transformed with these fusion plasmids. GFP fluorescence or β-galactosidase activity was monitored in the presence of EsrE overexpression or control plasmids. Among all the candidates, four showed significant differences from their controls. Three genes, including *murE* (UDP-*N*-acetylmuramoylalanyl-D-glutamate-2,6-diaminopimelate ligase), *murF* (D-alanyl-D-alanine-adding enzyme), and *sdhD* [succinate dehydrogenase (SDH), membrane protein SdhD] were activated, whereas another gene, *argI* (ornithine carbamoyltransferase chain I) was repressed by EsrE, as shown by the activated or repressed GFP fluorescence or β-galactosidase activities (**Figures 5A,B**) (Keseler et al., 2013). It has been reported that MurE and MurF were essential for cell growth by catalyzing the final two reactions of the biosynthesis of UDP-*N*-acetylmuramoyl-pentapeptide, the monomeric unit of peptidoglycan (Keseler et al., 2013). The strongest signal difference was detected between the strains that expressed the 5′ UTR of *sdhD*. The β-galactosidase activity in EsrE overexpressing strain was almost threefold that of its control. The *sdhD* gene encodes the membrane subunit of SDH, the primary dehydrogenase involved in aerobic electron transfer in *E. coli* (Keseler et al., 2013). Collectively, these findings implied that EsrE targeted multiple regulons to perform its function in *E. coli*.

### EsrE Is Required for Succinate Dehydrogenase Activity

**FIGURE 6:**
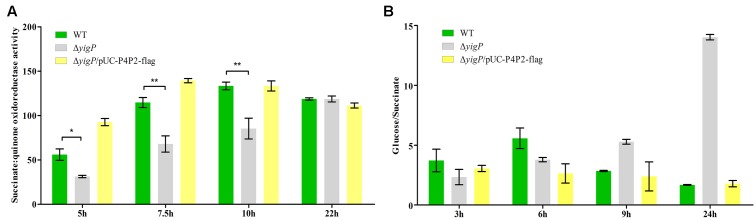
EsrE is required for succinate dehydrogenase activity. **(A)** Succinate dehydrogenase (SDH) activity assays were carried out on wild type, Δ*yigP*, and Δ*yigP* strains transformed with pUC-P4P2-flag at four different growth phases (5, 7.5, 10, and 22 h). Equivalent cell densities were collected, pelleted, and permeabilized by ultrasonication. The measurement of SDH activity was performed in triplicate using the Succinate Dehydrogenase assay kit (Nanjing Jiancheng Bioengineering Institute, China). The experiment was performed at least three times, and identical patterns were obtained. **(B)** Wild type, Δ*yigP*, and Δ*yigP* strains transformed with pUC-P4P2-flag were grown overnight in LB and then diluted to an OD_600_ of 0.03 in 100 mL of 0.2% minimal glucose or 0.4% minimal sodium succinate medium. Growth was monitored at 600 nm. The ordinate is the OD_600_ ratio. The experiment was performed at least three times, and identical patterns were obtained. The results of a representative experiment are shown. ^∗^*P* < 0.05, ^∗∗^*P* < 0.01.

SdhD is a membrane protein in the four-subunit enzyme complex of SDH (Keseler et al., 2013). SDH is an essential component of the tricarboxylic acid (TCA) cycle. It catalyzes the oxidation of succinate to fumarate and donates electrons to the electron transport chain (ETC; Cecchini, 2003). As described above, the expression level of *sdhD* was upregulated by EsrE. To further verify the relationship between EsrE and SDH, SDH activity was measured in wild type and Δ*yigP* strains at four different growth times. In the wild type strain, SDH activity doubled at 7.5 h, reached its peak at 10 h, and then plateaued (**Figure 6A**). In contrast, SDH activity of Δ*yigP* strain increased slowly during growth and reached its peak at 22 h (**Figure 6A**). The deletion of EsrE caused 40–50% decrease in SDH activity at 5, 7.5, and 10 h. The decrease could be rescued by EsrE *in trans* expressed by pUC-P4P2-flag (**Figure 6A**). The rapid increase of SDH activities from the later exponential phase to the stationary growth phase in wild type strain may be due to increasing amounts of EsrE, because its expression level boosted during the transition to the stationary phase (**Figure 1B**). The results indicated EsrE improved the activity of the SDH complex from the later exponential phase.

To further investigate the influence of EsrE on SDH activity, we replaced succinate with glucose in the minimal media. Reasoning that the predominant means of energy generation is glycolysis, SDH is essential only when the carbon source is succinate (Park et al., 1995). As expected, though the EsrE deletion mutant strain showed impaired growth on minimal succinate, it grew much better than the wild type strain on the glucose media (**Figure 6B** and Supplementary Figure S4). Meanwhile, the complementary expression of EsrE by pUC-P4P2-flag could rescue the growth defect on minimal media with succinate (**Figure 6B** and Supplementary Figure S4). Collectively, these findings indicated that EsrE was required for SDH activity in *E. coli*.

## Discussion

### The *yigP* Locus Encodes Two Products: EsrE and UbiJ

UQ is a redox-active lipid that is widely distributed in nature. The UQ structure has a conserved aromatic ring and an isoprenoid side chain of various lengths. The length of isoprenyl chain varies among species; there are six in *Saccharomyces cerevisiae*, eight in *E. coli*, and 10 in humans (Turunen et al., 2004; Bentinger et al., 2010; Nowicka and Kruk, 2010). Therefore, UQ in *E. coli* is designated Q_8_. The Q_8_ biosynthetic pathway is highly conserved and requires almost 11 genes, named *ubi* (Meganathan, 2001; Tran and Clarke, 2007). Q_8_ acts as a redox carrier in the plasma membrane, plays vital roles in aerobic respiration, oxidative stress adaptation, regulation of gene expression and other pathways dependent on the proton motive force (Aussel et al., 2014b). Recently, the *yigP* locus was renamed *ubiJ* that was characterized as an important element for Q_8_ biosynthesis in *Salmonella* and *E. coli* under aerobic conditions (Aussel et al., 2014a). Aussel et al. (2014a) believed that the aerobic growth defect was attributed to the blockage of Q_8_ biosynthesis due to mutant *yigP* locus. No sRNA was produced from the *yigP* locus because their frameshift mutant fragments at the C-terminal (the last 50 amino acids) coding sequence failed to rescue the growth defect (Aussel et al., 2014a). Though our results also showed that the *yigP* locus was required for Q_8_ biosynthesis in *E. coli*, we found that EsrE sRNA was also required for this process because in the absence of UbiJ, Q_8_ could still be produced in *E. coli* (**Figures 2**–**4**). Based on the evidence that the UbiJ deficient (frameshift) strain showed no phenotype and EsrE could only partially rescue the defect caused by 3′ UTR deletion at the *yigP* locus in aerobic growth, we inferred that not all the cellular Q_8_ content were required for aerobic growth of *E. coli*. Some minimal level (threshold level) of Q_8_ could be enough to maintain the high growth rate. This then led us to question whether the functions of EsrE and UbiJ were tightly related, and if there were advantages of hosting *esrE* within *ubiJ*. Our data showed that besides promoting Q_8_ biosynthesis, EsrE was also required for SDH activity, which suggests that the function of EsrE could be tied to UbiJ, and the two cooperated with each other in the respiratory chain of *E. coli*. However, more investigations are needed to verify this hypothesis.

### A New Insight into Gene Annotation: 3′ UTR-Embedded sRNAs

Ongoing studies reveal that sRNA genes are not limited to intergenic regions, as they are also detected in coding regions (Lalaouna et al., 2015; Miyakoshi et al., 2015a). These sRNAs might pose a problem for gene annotation because there can be more than one functional product from a single gene locus. One emerging class of such sRNAs is 3′ UTR sRNAs. Miyakoshi et al. (2015b) proposed that 3′ UTR sRNAs could be divided into two basic types according to their biogenesis. Type I sRNAs are transcribed from independent promoters within the coding region or the 3′ UTR of known genes. This type includes MicL (Guo et al., 2014), EsrE (described here) in *E. coli*, and DapZ in *Salmonella enterica* (Chao et al., 2012). Type II 3′ UTR sRNAs such as CpxQ (Chao and Vogel, 2016) and SorC in *S. enterica* (Miyakoshi et al., 2015a) strictly originate from the processing of the parental mRNAs. 3′ UTR sRNAs usually share partial sequences with their respective mRNA loci (Chao et al., 2012; Guo et al., 2014; Miyakoshi et al., 2015b; Chao and Vogel, 2016). Thus the phenotypes attributed to these loci may not depend solely on the protein. The functions of both products need to be considered. Moreover, the correlation between 3′ UTR sRNAs and their parental mRNAs are currently ambiguous since only a few examples have been studied. DapZ and MicL functions have been proven to have no relation with their parental mRNAs, while SorC and CpxQ mediate cross talk between their respective mRNAs (Chao et al., 2012; Guo et al., 2014; Miyakoshi et al., 2015a; Chao and Vogel, 2016). It seems that Type II sRNAs are more related to their parental mRNAs than Type I. Here we found that EsrE, a Type I sRNA, has four targets. Among them, three (*murE*, *murF*, and *sdhD*) were activated and one (*argI*) was repressed by EsrE (**Figures 5A,B**). These targets are functionally unrelated, which makes EsrE function more diversified thereby requiring more efforts to uncover. Besides, EsrE is important for cellular Q_8_ biosynthesis, but the corresponding targets and regulatory mechanisms are still unknown. Additionally, EsrE could activate *sdhD* to increase the levels of SDH, which connects the TCA cycle with the ETC. Thus, we anticipate that EsrE might have a vital role in regulating the respiratory chain of *E. coli*. Meanwhile, its respective mRNA, *ubiJ*, is also a key element of the aerobic ETC of *E. coli* (Aussel et al., 2014a). In general, Type I sRNA could cooperate with their parental mRNA. But still, the correlation of the parental mRNA function and targets of 3′ UTR sRNAs remain to be elucidated.

**FIGURE 7:**
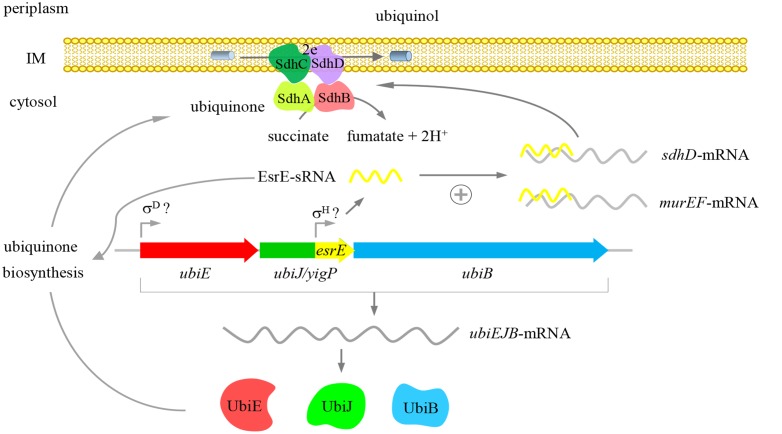
Proposed function of the *yigP* locus. The *yigP* locus may have two products, both of which are required for the aerobic growth of *E. coli*. One is a protein encoded by *ubiJ* that functions as a new factor involved in Q_8_ biosynthesis under aerobiosis. The other is sRNA transcribed from the 3′ region of the *yigP* locus and also required for Q_8_ biosynthesis as well as interacting with multiple targets involved in different cellular processes such as murein biosynthesis and the TCA cycle. This sRNA especially activates *sdhD* mRNA.

In summary, our study provided new evidence that the *yigP* locus had two products, the UbiJ protein and EsrE sRNA. Both products were essential for Q_8_ biosynthesis and further contributed to aerobic growth of *E. coli* (**Figure 7**). Our results also showed that EsrE might target multiple mRNAs, including *murE*, *murF*, *sdhD*, and *argI*, which are involved in different cellular processes. Furthermore, our data indicated EsrE was required for the activity of SDH in succinate-dependent growth of *E. coli*. Additionally, our characterization of EsrE indicated that sRNAs (sRNAs from UTRs) may be masked in protein-coding regions. Phenotypes generally caused by deficiencies in protein may also be due to uncovered sRNA defects.

## Author Contributions

HW and HZ conceived and designed the experiments. HX, XY, and QT performed the experiments. JY contributed reagents/materials/analysis tools. HX, HW, and HZ analyzed and wrote the paper.

## Conflict of Interest Statement

The authors declare that the research was conducted in the absence of any commercial or financial relationships that could be construed as a potential conflict of interest.
