# Supplementary material for: EsrE-A yigP Locus-Encoded Transcript-Is a 3′ UTR sRNA Involved in the Respiratory Chain of E. coli
Source: Front Microbiol. 2017 Aug 29;8:1658. doi: 10.3389/fmicb.2017.01658 (PMC5581919; doi:10.3389/fmicb.2017.01658)
Supplement: Supplementary file 1 [file Data_Sheet_1.docx]

Supplementary Material

EsrE⎯a *yigP* locus-encoded transcript⎯is a 3’ UTR sRNA involved in the respiratory chain of *E. coli*

Hui Xia^1^, Xichen Yang^1^, Qiongwei Tang^1^, Jiang Ye^1^, Haizhen Wu^1,2*^, Huizhan Zhang^1,2*^

*** Correspondence:** Haizhen Wu: wuhzh@ecust.edu.cn, Huizhan Zhang: huizhzh@ecust.edu.cn

# Table S1 Strains and plasmids used in this study

| Strain or plasmid | Relevant genotype | Source or reference |
| --- | --- | --- |
| Strains | | |
| *E. coli* | | |
| JM83 | wild-type | Laboratory stock |
| Δ *yigP* | Δ yigP::Am of wild-type | This study |
| Δ *yigP-3’end* | Δ 153 bp in 3’ end of *yigP* locus::Am of wild-type | This study |
| Δ *yigP-5’end* | Δ 283 bp in 5’ end of *yigP* locus::Am of wild-type | This study |
| WT::*flag* | *yigP*::linker-3×Flag of wild-type | This study |
| Plasmids | | |
| pUC-P4P2 | pUC18 derivative carrying the 367 nucleotides located in the 3’ end of *yigP* locus ( Ap^r^ ) | Laboratory stock |
| pUC-P43L9 | pUC18 derivative carrying the 267 nucleotides located in the 3’ end of *yigP* locus ( Ap^r^ ) | Laboratory stock |
| pUC-yigP-flag | pUC18 derivative carrying the 241bp sequences upstream *yigP* locus and 606 nucleotides of *yigP* locus and a linker-3×Flag fusion before the stop codon ( Ap^r^ ) | This study |
| pUC-P4P2-flag | pUC18 derivative carrying the 367 nucleotides located in the 3’ end of *yigP* locus and a linker-3×Flag fusion before the stop codon ( Ap^r^ ) | This study |
| pUC-P43P2-flag | pUC18 derivative carrying the 313 nucleotides located in the 3’ end of *yigP* loci and a linker-3×Flag fusion before the stop codon ( Ap^r^ ) | This study |
| pMAK-yigP-flag | pMAK705 derivative carrying *yigP* and a linker-3×Flag fusion before the stop codon ( Cm^r^ ) | This study |
| pT-002 | pDM18-T derivative containing P43P2 fragment with delete mutation in site 1 ( Ap^r^ ) | Laboratory stock |
| pT-005 | pDM18-T derivative containing P43P2 fragment with delete mutation in site 2 ( Ap^r^ ) | Laboratory stock |
| pT-003 | pDM18-T derivative containing P43P2 fragment with delete mutation in site 3 (Ap^r^) | Laboratory stock |
| pT-004 | pDM18-T derivative containing P43P2 fragment with delete mutation in site 4 ( Ap^r^ ) | Laboratory stock |
| pT-P4P2 | pMD18 derivative carrying the 367 nucleotides located in the 3’ end of *yigP* locus ( Ap^r^ ) | This study |
| pT-NS | pMD18 derivative carrying the 367 nucleotides of nonsense fragment ( Ap^r^ ) | This study |

## Table S2 Primers and probes used in this study

| Primer | Sequence (5’ to 3’) |
| --- | --- |
| Construction of *gfp* translational fusions | |
| dnaX-F | TATAATGCATTCGTGAATCCACCTTCCA |
| dnaX-R | GCTAGCTAGCATGATGAATACGCCCTAA |
| serS-F | TATAATGCATGTATCCCTGTGGTCGCAG |
| serS-R | GCACGCTAGCTACTTTACGACGCTCTTC |
| fldA-F | TATAATGCATATCCGTGGGCAATTTTCC |
| fldA-R | TATAGCTAGCGTCATGGACATCGGCAAC |
| asnS-F | TATAATGCATCCCCCCATTTACGGGTG |
| asnS-R | TGTAGCTAGCATCAAAGCAGGAACCGTC |
| accD-F | GAGCATGCATCATTCATGGTCTGTTG |
| accD-R | TATTGCTAGCCGGACAGACCTCAAGATT |
| argI-F | GAGCATGCATAATTCATTGAGGCGTTAG |
| argI-R | TATAGCTAGCTTCGAAGATGAGCGCGAT |
| pyrG-F | GAGCATGCATCTCCTGATTTCAATAGTG |
| pyrG-R | ATTGACGCTAGCGTACGGATCCAGTTT |
| folD-F | AAATATGCATATCCCCTTCGATCTACGTAACAGAT |
| folD-R | ACACACGCTAGCTTTGCTTGCGACATAAATT |
| plsC-F | GAGCATGCATTGATTTCAGGTGACGTACAA |
| plsC-R | TCCTCCTTTGCTAGCGAATACACAGACTAAGATGC |
| holB-F | GAGCATGCATGCAGCACAAGATAAAAGC |
| holB-R | TATACGCTAGCCATCAACTGACATCCACG |
| ftsX-F | TATAATGCATTTGATGGCAACGCACGAC |
| ftsX-R | ATATAGCTAGCTGCGCCGTGGAAGGCATA |
| rnpA-F | GAGCATGCATCAACCGTCTGTACTGAAG |
| rnpA-R | TATAGCTAGCTTCATGGGCGCGTCGAAC |
| pheT-F | TAGCATGCATTACTCTGGTTTCGCCTTC |
| pheT-R | CGCTAGCTAGCGACATTCACTTTTGTCAC |
| tdcF-F | GAGCATGCATTATCCTAACCTGACAATCC |
| tdcF-R | TATAGCTAGCCTTGATGATATCGCCCACG |
| murE-F | GCGCATGCATGTTGTTGTTATCAACGAT |
| murE-R | TATAGCTAGCTCGCGAAGGTGCGTCTG |
| ftsA-F | CCTTATGCATATGAAACGTTTGGCTCGC |
| ftsA-R | GCTAGCTAGCGATATTGACCATACCGTC |
| ftsW-F | TATAATGCATCCGGAAGTGGCAGAACAA |
| ftsW-R | ATATGCTAGCCATGATAAAGCCAATCGC |
| murF-F | TATAATGCATCATGCCAAAGTGATGGAA |
| murF-R | TACTAGCTAGCCGTCAGTTTTCGGGTATC |
| murG-F | TAGCATGCATCTGATTATGTCGACAGCCAT |
| murG-R | CGACGCTAGCTGCTTTTATACCTTTTCC |
| Construction of *lacZ* translational fusions | |
| cbtA-F | GAGCATGCATTATGCAAAAGGGCTGACC |
| cbtA-R | TCACGCTAGCCAGCAGTCGGGACAGCA |
| sdhD-F | GAGCATGCATCATCCTTACCGCTCTGGC |
| sdhD-R | TCACGCTAGCGATGTAGAGCGTCAGGAC |
| hflC-F | GAGCATGCATGGTGGTAACGCCCCTG |
| hflC-R | TCACGCTAGCCGTAATACCGCGCTCACC |
| fliZ-F | GAGCATGCATATCGAAACGTTGCCGGAG |
| fliZ-R | TCACGCTAGCTAATTTACGGCAATGCGC |
| mioC-F | GAGCATGCATAGCGCCAAAGACTACCCTTCC |
| mioC-R | TGTCACGCTAGCCAGCTTTTCAGCCAGGTGTTC |
| Chromosomal mutation | |
| yigP-QC1 | CGCTGCATCGTGGTTATAAGTTCTGA  CAGGAGACCGGAAATTCCGGGGATCCGTCGACC |
| yigP-QC2 | CTGGCGTCATTTAGCCTCCAGTTTTTCCAGC  CGTTTGGTCTGTAGGCTGGAGCTGCTTC |
| yigP-QC3 | CGCGGAGGCGCAAAGTTCCTGCATCACGG  CATTAAGTGAATTCCGGGGATCCGTCGACC |
| yigP-QC4 | CTGGCGTCATTTAGCCTCCAGTTTTTCC  AGCCGTTTGGTCTGTAGGCTGGAGCTGCTTC |
| Construction of Flag-fusion plasmids | |
| P4-F | TCGCTATTACGCCAGCAGCTTACCGCACTGATTC |
| P2-R | GATTCATTAATGCAGCGTACTTCACCTGGCGTC |
| P43-F | TCGCTATTACGCCAGGTGGTGCAAAACTTCGTTG |
| yigP-F | CGGGATCCGAGCCGCTGAGCAAAGCCTA |
| yigP-R | AACTGCAGTGTTAGTGCCGTTTTTCTCC |
| Flag-F | CGACTACAAGGACCACGACATCGACTACAAGG  ACGATGACGACAAGTGACGCCAGGTGAAGT |
| Flag-R | TCGATGTCGTGGTCCTTGTAGTCGCCGTC  GTGGTCCTTGTAGTCTTTAGCCTCCAGTTTTTC |
| Linker-F | GTTCAGGCGGAGGTGGCTCTGGCGGTG  GCGGTAGTGACTACAAGGACCACGACGG |
| Linker-R | CGCCAGAGCCACCTCCGCCTGAAC  CGCCTCCACCTTTAGCCTCCAGTTTTTC |
| primers for qRT-PCR | |
| rpoD-RT-F | CAGCCAGGTTCAATGCTCCGTT |
| rpoD-RT-R | TTCCTGGGAAAGCTCAGAACCGA |
| ubiE-RT-F | GTTTTCCATTCCGTGGCATCA |
| ubiE-RT-R | AGGCGGGAGAATTTCGCTGT |
| yigP-S1 | CTGCTGGGTAAAGTATTGCG |
| yigP-S2 | GCGAATCAGTGCGGTAAGC |
| yigP-X1 | AATCAGCAAAGCCATGCG |
| yigP-X2 | TCAGGGCATCAACAGCAC |
| ubiB-RT-F | GGGATGATGGTGATGGAGCG |
| ubiB-RT-R | CGAAGATGTTGCCAGGGTGC |

## Table S3 Overview of EsrE predicted targets verified in this study

| Target gene | Product description^a^ | Insert  5’ end^b^ | Fused  codon | Fusion  vector^c^ | prediction algorithm |
| --- | --- | --- | --- | --- | --- |
| *dnaX* | DNA polymerase III, γ subunit | -36 | 39 | pXG-10 | CopraRNA |
| *serS* | seryl-tRNA synthetase | -63 | 39 | pXG-10 | CopraRNA |
| *fldA* | flavodoxin 1 | -56 | 35 | pXG-10 | CopraRNA |
| *asnS* | asparaginyl-tRNA synthetase | -59 | 48 | pXG-10 | CopraRNA |
| *accD* | acetyl-CoA carboxyltransferase, β subunit | -89 | 47 | pXG-10 | CopraRNA |
| *argI* | ornithine carbamoyltransferase chain I | -33 | 53 | pXG-10 | CopraRNA |
| *pyrG* | CTP synthase | -131 | 44 | pXG-10 | CopraRNA |
| *folD* | 5,10-methylene-tetrahydrofolate dehydrogenase / 5,10-methylene-tetrahydrofolate cyclohydrolase | -40 | 54 | pXG-10 | CopraRNA |
| *plsC* | 1-acylglycerol-3-phosphate  O-acyltransferase | -50 | 20 | pXG-10 | CopraRNA |
| *holB* | DNA polymerase III, δ prime subunit | -107 | 68 | pXG-30 | CopraRNA |
| *ftsX* | putative transport protein, ABC superfamily | -91 | 63 | pXG-30 | CopraRNA |
| *rnpA* | RNase P protein component | -142 | 61 | pXG-30 | CopraRNA |
| *pheT* | phenylalanyl-tRNA synthetase β-chain | -131 | 69 | pXG-30 | CopraRNA |
| *tdcF* | predicted enamine/imine deaminase | -121 | 73 | pXG-30 | CopraRNA |
| *murE* | UDP-N-acetylmuramoylalanyl-D-glutamate 2,6-diaminopimelate ligase | -169 | 20 | pXG-30 | CopraRNA |
| *ftsA* | essential cell division protein FtsA | -179 | 35 | pXG-30 | CopraRNA |
| *ftsW* | essential cell division protein FtsW;  lipid II flippase | -173 | 62 | pXG-30 | CopraRNA |
| *murF* | D-alanyl-D-alanine-adding enzyme | -176 | 35 | pXG-30 | CopraRNA |
| *murG* | N-acetylglucosaminyl transferase | -89 | 72 | pXG-30 | CopraRNA |
| *cbtA* | CP4-44 prophage; toxin of the CbtA-CbeA toxin-antitoxin system | -181 | 30 | pXH-LacZ | TargetRNA 2.0 |
| *sdhD* | succinate:quinone oxidoreductase, membrane protein SdhD | -155 | 30 | pXH- LacZ | TargetRNA 2.0 |
| *hflC* | regulator of FtsH protease | -182 | 31 | pXH- LacZ | TargetRNA 2.0 |
| *fliZ* | FliZ DNA-binding transcriptional regulator | -210 | 31 | pXH- LacZ | TargetRNA 2.0 |
| *mioC* | flavoprotein involved in biotin synthesis | -317 | 26 | pXH- LacZ | TargetRNA 2.0 |

^a^Product description are annotated according to EcoCyc

^b^5’ end of the target gene insert relative to annotated ATG

^c^pXG-10 and pXG-30 were describe previously(Urban and Vogel, 2007), pXH-LacZ was constructed in this study based on pXG-30 where the *gfp* gene replaced by *lacZ* gene.


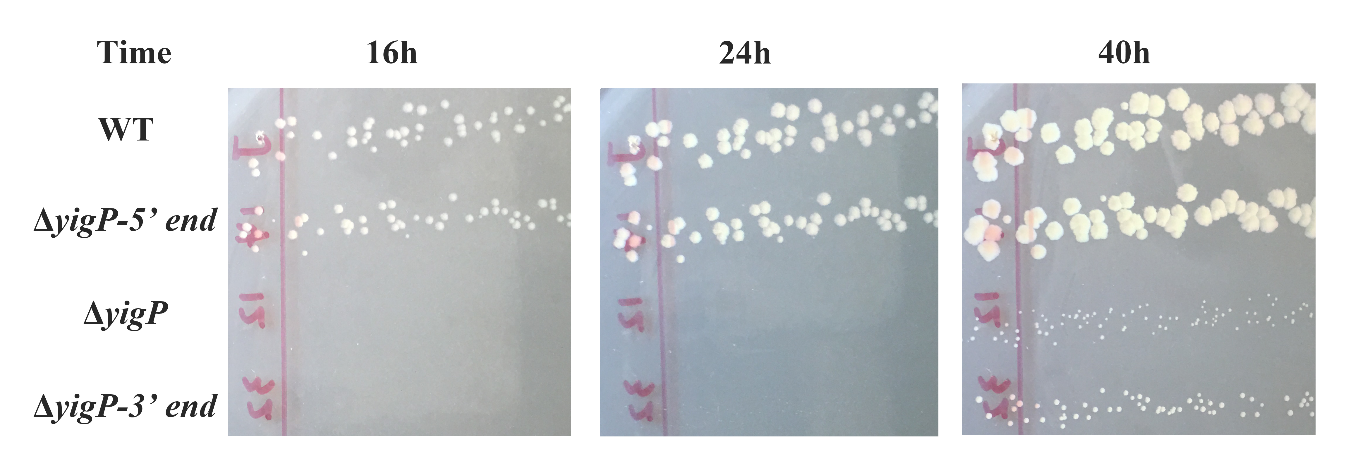


**Supplementary Figure 1.** The important region of *yigP* locus is located at 3’ end. Three different mutant strains Δ*yigP*, Δ*yigP*-*5’end* and Δ*yigP-3’end*, along with wild type strain were grown on LB plates for 16, 24 and 40 h at 37°C.

**
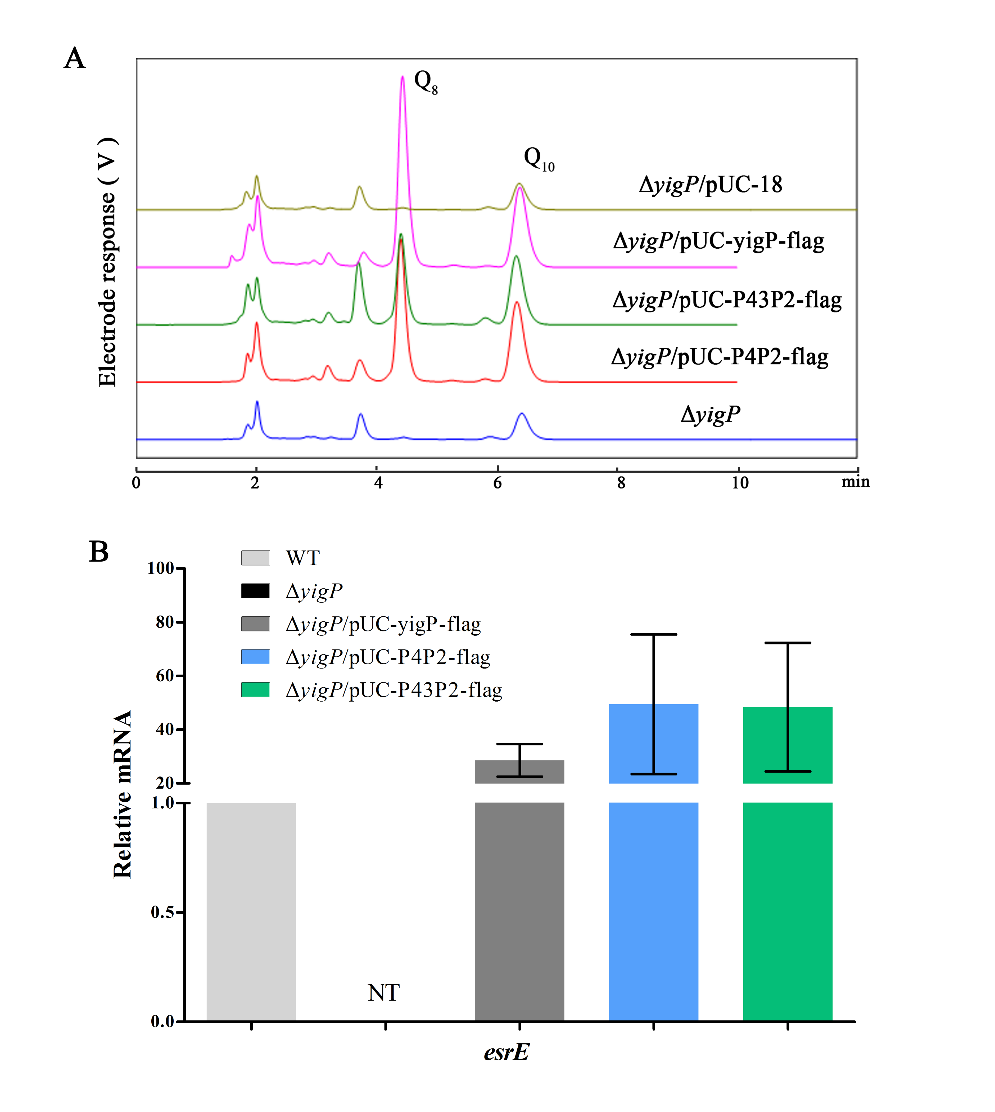
**

**Supplementary Figure 2.** The 3’ region of *yigP* locus transcribes a product. A) HPLC separation of Δ*yigP* and Δ*yigP* transformed with pUC-yigP-flag, pUC-P4P2-flag, pUC-P43P2-flag and pUC18 with the eluate analyzed with A_275_. The identified quinones Q_8_ and Q_10_ are indicated. B) qRT-PCR analysis of 3’ region transcripts of wild-type, Δ*yigP* and Δ*yigP* transformed with pUC-yigP-flag, pUC-P4P2-flag and pUC-P43P2-flag at 5 h.


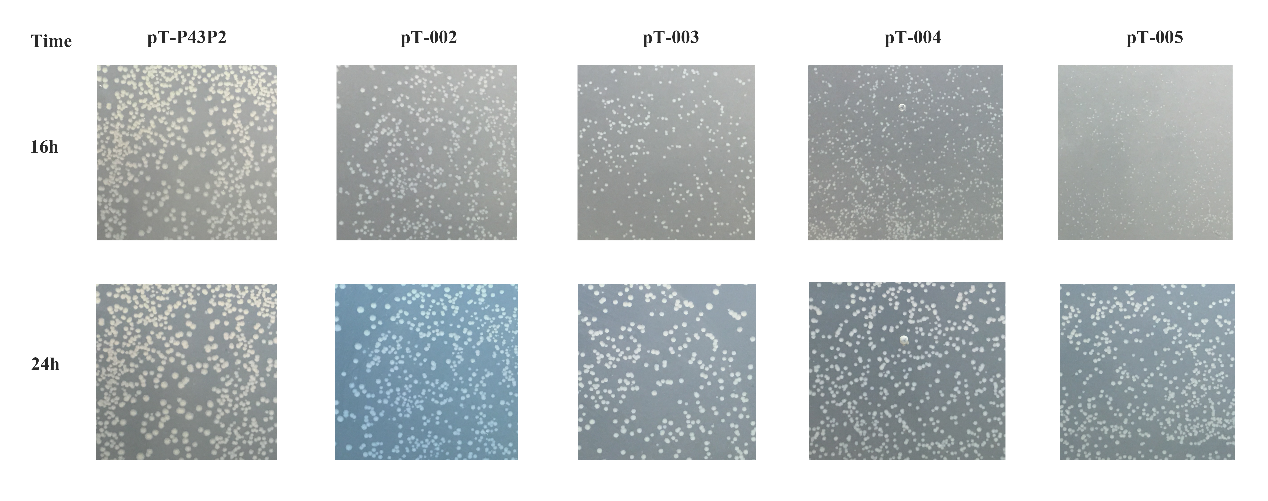


**Supplementary Figure 3.** Four site mutants fragments all have a certain extent affection of the rescue ability of P43P2 fragments. The Δ*yigP* strains with pT-P43P2, pT-002, pT-003, pT-004 and pT-005 were grown on LB plates for 16 and 24 h at 37°C.


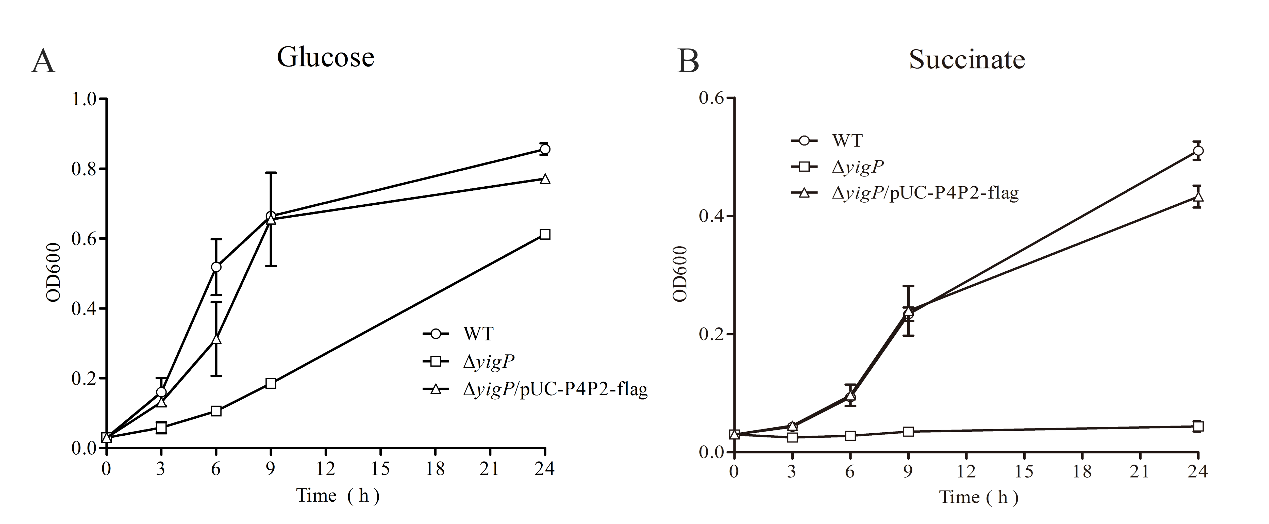


**Supplementary Figure 4.** EsrE is an important element for growth on minimal medium, especially for minimal sodium succinate medium. A) Wild-type, Δ*yigP* and Δ*yigP* transformed with pUC-P4P2-flag were grown overnight in LB and then diluted at an OD_600_ of 0.03 in 100 mL of ( 0.2 % ) minimal glucose medium respectively. Growth was monitored at 600 nm. The experiment was performed at least in triplicate, identical patterns were obtained, and results of a representative experiment were shown. B) Wild-type, Δ*yigP* and Δ*yigP* transformed with pUC-P4P2-flag were grown overnight in LB and then diluted at an OD_600_ of 0.03 in 100 mL of ( 0.4 % ) minimal sodium succinate medium respectively. Growth was monitored at 600 nm. The experiment was performed at least in triplicate, identical patterns were obtained, and results of a representative experiment were shown.

Urban, J.H., and Vogel, J. (2007). Translational control and target recognition by *Escherichia coli* small RNAs *in vivo*. *Nucleic Acids Res* 35(3)**,** 1018-1037. doi: 10.1093/nar/gkl1040.
